# Mindful Self-Compassion as an Antidote to Burnout for Mental Health Practitioners

**DOI:** 10.3390/healthcare11202715

**Published:** 2023-10-12

**Authors:** T. Richelle Lyon, Anne Galbraith

**Affiliations:** 1 Lyon Research & Consulting LLC, Kerrville, TX 78028, USA; 2 Marriage and Family Therapist, Los Angeles, CA 90025, USA; anne.galbraith@gmail.com

**Keywords:** burnout, mental health practitioner, self-care, self-compassion

## Abstract

The objective of this correlational study was to explore the relationship between levels of self-compassion and burnout for currently practicing mental health practitioners (MHPs) in the United States. All professionals are vulnerable to burnout based on various types of organizational stressors, but burnout is of particular concern for health care service providers who may need to adopt a stance of detachment, or emotional distance, as relief from intense workloads, with clients. The data were collected through an online survey. Regression analysis found that scores from Neff’s Self-Compassion Scale were a significant negative predictor of levels of MHP burnout, as assessed by Schaufeli et al.’s Burnout Assessment Tool, *p* < 0.001. The implication of this finding is that cultivating self-compassion appears to be a pragmatic self-care strategy for MHPs to mitigate the negative effects of burnout. More educational and occupational training in self-compassion practices as self-care should be provided to help protect the physical and emotional well-being of MHPs. The deleterious systemic effects of burnout make MHP self-care an ethical issue, along with the need to identify protective factors, prevention, and treatment of burnout.

## 1. Introduction

### 1.1. Burnout

In 2019, the World Health Organization included occupational burnout in the 11th Revision of the International Classification of Diseases and classified it as a phenomenon related to prolonged exposure to chronic, unsuccessfully managed workplace stress [1]. The term burnout first appeared in the academic literature in the 1970s as a social psychology construct, resulting from research conducted by pioneers in industrial organizational psychology who were interested in job stress [2,3,4]. To develop the original theory, qualitative exploratory research was conducted in the human health care services industry, due to its association with emotion and interpersonal stressors [5,6]. This led to a three-dimensional theoretical framework of burnout [2] which, decades later, remains the gold standard for conceptualizing it [7].

Burnout is widely accepted as a unique multidimensional psychological construct [8]. It is a maladaptive response to occupational stressors that, when left untreated, may decrease well-being by negatively affecting both mental and physical health [9]. Burnout also causes persistent interpersonal and emotional strain [2] since it is not only an individual experience, but also carries social implications related to conceptions of the self and others [10]. It is situation-specific (i.e., work-related) [4] and not generally associated with psychopathology or other types of organic illness [2,11]. 

In recent years, an updated and rigorously tested conceptualization of burnout has emerged [12]. This has confirmed that burnout is a single unified construct, but with core symptoms categorized across four dimensions, that are all related to occupational performance [13]. Exhaustion is severe energy depletion, defined as feeling physically tired, mentally drained, and worn-out to the extent that the regulation of cognitive and emotional processes is impeded [13]. Cognitive impairment is reduced performance due to difficulties with memory, attention, and concentration [13]. Emotional impairment is feeling overwhelmed by intense emotional reactions, often expressed as frustration, irritability, anger, or feeling sad at work without the ability to control emotions [13]. Mental distance is an attempt to decrease exhaustion through psychological detachment from work, denoted by indifference and cynicism [13]. These four dimensions constitute the primary domains of burnout, along with two sets of secondary burnout symptoms, psychological distress, which is a nonphysical complaint like difficulty sleeping, excessive worrying, or feeling tense and anxious; and psychosomatic distress, which is a physical complaint like chest or stomach pain or headaches that are exacerbated by a psychological problem and not attributable to a physical disorder [7].

All professionals are vulnerable to burnout based on various types of organizational stressors, but burnout is of particular concern for health care service providers who may need to adopt a stance of detachment, or emotional distance, as relief from intense workloads, with clients [2]. This is especially true for mental health practitioners (MHPs), a population of workers defined as persons with current, appropriate licensure in a mental health profession who are permitted to evaluate and care for patients within the scope of professional practice [14]. Burnout has a high prevalence for psychotherapists [15]. It is estimated that burnout affects 40% of MHPs, based on a systematic review of 62 studies that presented data on the “prevalence and determinants of burnout” for MHPs across 33 countries [16]. 

The risk of developing burnout can begin early in one’s career, as soon as during an internship [3,17], due to external factors like unrealistic workloads [18], lack of professional training, stressful work environments [1], or inconsistent expectations about work performance, in addition to the emotional demands of the profession [19]. Internal risk factors for burnout include age, years of experience, personal characteristics, and coping styles; thus, younger or less experienced MHPs may not have the training or resources available to help meet the challenges of the profession [6,20]. Additional contributing factors include perfectionism, anxiety, low self-efficacy, and interpersonal characteristics like shyness [12,21]. Although personal vulnerability and/or problems outside of work may facilitate the development of burnout, research demonstrates that it is tied etiologically to an imbalance between high job demands and insufficient job resources [22]. It is an individual reaction to persistent work stress and professional demands, with a progressive developmental trajectory, as opposed to being simply a personal problem [23].

The effects of burnout are concerning for health care service providers, since empathy (without sympathetic emotional distress) and compassion are critical facets of high-quality patient care and are closely related to feelings of work satisfaction and meaning [24,25,26]. For example, compassion satisfaction, the opposite of compassion fatigue, is the pleasure or positive effects experienced when helping people cope with suffering, and the perception that one’s work is contributing positively to society [20]. MHPs are particularly vulnerable to compassion fatigue (a relational source of stress associated with helping others) [5] and burnout since the very nature of their work is to help clients recover from the effects of trauma and suffering [27]. Burnout is also consistently related to poor employee retention, higher service costs, and suboptimal client care [26,28].

### 1.2. Self-Care

Self-care is a known antidote to burnout because it is a preventive action, intended to promote well-being, harmony, and balance in a person’s life [29,30]. Self-care is the ability to maintain personal health, prevent disease, and cope with illness or disability either with, or without, a supportive health-care provider [31]. It is used by MHPs to nurture the self, manage anxiety, and maintain optimal performance [32]. Domains of self-care practice include actions that promote physical health, social support, and spirituality [33]. Self-care is a necessity for all employees in the helping professions [33,34], but it is considered essential for MHPs who are ethically obligated to assume responsibility for preserving personal health [31]. 

Most MHPs can skillfully articulate the benefits of self-care to their clients, even as they lack vigilance regarding their own [35,36]. Research has repeatedly revealed that many MHPs personally experience a disconnect between knowledge of self-care and utilizing personal self-care actions [37]. This may be due in part to being unskilled at personal self-care strategies that reduce burnout [28,38] or simply as there are barriers that prevent them from taking the necessary steps to mitigate it [39]. The demands of the profession can create challenges due to a lack of training, resources, supervision, time, or finances [40]. Fortunately, there is growing interest in promoting self-care strategies during MHP training programs and in mental health work settings [35,36,41]. This may address the urgent need to identify risk factors and protective measures to help protect MHPs’ psychological well-being [28,42,43], especially by educating MHPs about self-care actions that are within their individual control. 

### 1.3. Mindful Self-Compassion Practices as Self-Care

Research has shown that mindfulness-based practices are effective in treating burnout because they help improve therapeutic and stress-management skills by instructing individuals on how to shift their perspective and cope adaptively [37]. Mindfulness is the moment-to-moment awareness of experience, coupled with acceptance and a non-judgmental attitude [44,45]. Benefits of mindfulness-based practices include improvements to self-efficacy [46], job satisfaction and resilience [18,47], burnout prevention [29,48], and self-awareness through self-compassion [49,50,51]. Therapists’ self-awareness is critical since it is correlated with resilience, which helps defend against burnout [52]. 

Self-compassion practice, as a component of mindfulness, is rooted in the principles of Buddhist psychology [53]. According to this framework, self-compassion is a function of a caring, kind, and nonjudgmental attitude toward oneself especially as related to feelings of inadequacy and failure [53]. Self-compassion practices appear to be effective in alleviating burnout when incorporated as self-care [50], and self-compassion training reduces burnout symptoms [54]. Adopting a mindful, self-compassionate perspective is beneficial for MHPs not only because it helps regulate negative emotions, but also because it may kickstart positive feelings toward oneself without the need to falsely inflate self-concept or to avoid or repress feelings that are painful [53]. Self-compassion means accepting the reality that failure and disappointment are natural parts of the human condition, which negates the need to evaluate personal performance in relation to others or ideal standards [53]. In turn, this may lead to a greater sense of compassion toward others [53,55,56] while boosting emotional resilience [57,58], personal resilience [59], and clinician resilience [60]. Randomized follow-up studies have shown the positive effects of mindfulness training on coping skills, self-care, and stress management as many as six years later [61]. There is abundant research on the benefits of mindful self-compassion for other types of healthcare workers [62], including protection from the negative effects of burnout [31,35]; but the potential of self-compassion practices for negating MHP burnout is understudied. That is the gap that this study has addressed. Research in this domain is especially needed because of the potential of self-compassion practices to be a practical type of self-care that is well-suited as an individual, accessible, and cost-effective preventive action. Therefore, the research question for this correlational study was, what is the effect of self-compassion on burnout for currently practicing MHPs in the United States?

## 2. Materials and Methods

### 2.1. Study Design and Sample

Recruitment was conducted using snowball sampling on social media platforms (e.g., LinkedIn, Facebook, Alignable, Twitter) and email. A direct URL/hyperlink to the survey was embedded in the invitation to participate, along with a request to forward it to potentially willing respondents. Participants were assured anonymity. Inclusion criteria for the study were adults aged 18 or older, currently practicing as an MHP in the United States, defined as a population of workers with appropriate licensure in a mental health profession who are permitted to evaluate and care for patients within the scope of professional practice [14]. According to the a priori G*Power analysis, the minimum recommended sample size was *n* = 107 to reach an actual power of 0.9519, with a critical *F* = 3.08371, using parameters for linear regression *F*-test, fixed model, with effect size (0.015), power (0.90), alpha (0.05), and *R*^2^ deviation from zero [63,64].

### 2.2. Instrument

A 69-item online survey was utilized to collect data. The survey began with the statement of informed consent and items to determine eligibility. Qualified respondents were then directed to 7 demographic questions to help understand participants’ background characteristics: (a) their type of MHP license, (b) gender identity, (c) level of education, (d) years of experience in practice, (e) average number of hours worked per week, (f) the amount of time spent in different work settings (e.g., private practice, agency, community health center, or hospital), and (g) the percentage of their total work time spent delivering mental health services in person or through telepsychology. This was followed by the 26 self-compassion items and the 33 BAT items to assess burnout.

#### 2.2.1. Self-Compassion

Self-compassion was measured using the 26-item Self-Compassion Scale (SCS) [65]. The SCS is an accurate, empirical instrument that provides a single-construct measure of self-compassion that is appropriate for assessing psychological outcomes related to varying levels of self-compassion [53,65]. This measure includes 6 highly intercorrelated theoretically based subscales that quantify the following distinct, opposing dimensions of self-compassion: self-kindness vs. self-judgment, common humanity vs. isolation, and mindfulness vs. over-identification [53,65]. 

The SCS has undergone extensive psychometric testing and is determined as valid and reliable [65]. Scale items were developed from pilot testing with multiple phases of small focus groups. Open-ended questions related to self-compassion were explored to identify ordinary language about reactions to painful experiences, failure, and group members’ notions of self-compassion. A pool of potential items was created from feedback regarding item comprehensibility and relevance, and then administered to a large sample for testing reliability and factor loadings. Convergent validity was assessed against previously established scales with related constructs, and final items were selected based on exploratory and confirmatory factor analysis [65]. Construct validity was tested by assessing correlation coefficients for the SCS against other scales measuring similar constructs [65]. Internal reliability ratings consistently surpass Nunnally’s standard [66] including when they are tested on large multicultural samples, such as university students in Tehran, Iran (α = 0.78 to 0.93) [67] and China (α = 0.84) [68]. Per the instructions [65], participants in this study were asked to read each statement carefully before indicating how often they behave in the stated manner on a scale from 1 (*Almost never*) to 5 (*Almost always*). Full-scale scores were computed as the grand mean of the 6 subscales, after reverse coding for the 13 items related to self-judgment, isolation, and over-identification [65]. Higher values indicate more self-compassion. The SCS is available for use in research, clinical, and teaching settings without special permission [65]. Cutoff values to assess levels of self-compassion are as follows: 1–2.5 (low), 2.5–3.5 (moderate), and 3.5–5.0 (high) [65].

#### 2.2.2. Burnout 

Burnout was measured with the 33-item Burnout Assessment Tool (BAT) [7]. The BAT was developed in multiple stages. First, a provisional measure was created from in-depth interviews and analyses of the reliability and validity of existing burnout questionnaires, followed by pilot studies to inform the scoring procedures [7]. Extensive psychometric analyses resulted in 6 subscales that assess 4 strongly interrelated core dimensions of burnout, with 50–64% of shared variance (e.g., exhaustion, mental distance, cognitive impairment, and emotional impairment) and two secondary dimensions (e.g., psychological complaints and psychosomatic complaints) [7]. Employees with the highest burnout scores were dissatisfied with their work, had more health complaints, more job demands, and fewer job resources. Furthermore, a trend was detected whereby high scores were consistent across scales, such that those who were classified as “high” or “low” on one subscale tended to have high scores across all subscales, which is a further indication that total BAT scores can be used to indicate burnout [7]. An essential utility of the BAT is its usefulness in helping organizations screen for burnout levels, either to identify individuals or groups to which specific preventive measures may be targeted or to estimate current rates of employee burnout [7].

The BAT has undergone extensive psychometric testing on data from large, representative Dutch and Flemish samples (*n* = 1500 each) from the working population [7]. Overall, the instrument and its 4 core subscales have demonstrated excellent convergent and discriminant validity and internal consistency, with α = 0.96 (Flanders sample) and α = 0.97 (Netherlands sample) [7]. Test–retest reliability was good, despite some variation, with satisfactory stability coefficients. Inter-rater reliability was low to moderate as self-reported scores differed slightly from peer ratings; however, it surpassed findings for the MBI (except for cognitive impairment) [7]. Total BAT scores may be used as an individual or organizational measure to indicate burnout levels overall or each dimension may be assessed independently to provide further specifications of individual burnout [7]. The authors recommend utilizing the following clinically validated cutoff values for diagnostic use as a point of reference to assess individual burnout risk [13]: Green (<2.58; indicates no burnout), Orange (≥2.59 and <3.02; indicates risk of burnout), and Red (≥3.02; indicates likely burnout) [7]. For this study, participants were asked to rate each BAT statement on a scale from 1 (*Never*) to 5 (*Always*) according to how often each applied to their experience of their work situation [7]. Scores were calculated by computing means for all items (full or subscale). No BAT items require reverse coding. Higher values indicate more burnout symptoms [7]. 

### 2.3. Procedure

Data were collected from 22 April 2022 to 14 June 2022 through an online survey hosted on Survey Monkey, with settings to ensure that no identifying information, including IP address, was collected. No incentive was offered, other than a statement that even though participants could not expect any direct benefit from the study, the information collected would possibly provide general benefits to MHPs by contributing knowledge about factors that affect professional burnout. 

### 2.4. Ethical Consideration

Prior to data collection, approval for conducting research with human subjects was received from California Southern University’s Institutional Review Board (Re: IRB#210422-76357), in concordance with university policies and the Declaration of Helsinki and the Ethics and Clinical Research Committee. Participants were provided with sufficient information to allow informed consent, including a brief description of the study’s purpose and procedures, their right to voluntarily decline or withdraw from the study at any time, and any known potential risks, discomfort, adverse effects, or benefits. Participants were assured full anonymity and provided with contact information to direct questions regarding their rights as a research participant or about their experience, including any adverse effects as a result of participating in the study per the Ethics Code of the American Psychological Association [69].

### 2.5. Data Analysis

Survey responses were downloaded from Survey Monkey to IBM SPSS Statistics (version 28.0.0) [70] for a series of statistical procedures. First, the data were reviewed to eliminate incomplete responses and to apply reverse coding to the self-judgment, isolation, and over-identification subscales [65]. Next, two new variables were computed for the standard deviation of the 26 SCS items and 33 BAT items, to identify non-engaged respondents. When responses across a scale from a single respondent do not vary, it may indicate non-engagement in the survey, which can negatively impact the reliability or validity of the results [71]. No standard deviations of 0 were detected and no cases were removed for non-engagement. Descriptive statistics were then generated for the 7 demographic variables and the SCS and BAT survey items, followed by reliability statistics (Cronbach’s α) to assess the internal reliability of the scales.

Since the aim of this study was to explore the relationship between self-compassion and burnout, the primary analysis (Model 1) consisted of linear regression with SCS scores as a continuous numeric predictor variable and total BAT scores as a continuous numeric criterion variable. Three additional numeric variables representing participant characteristics (e.g., years of experience, average number of hours worked per week, and percentage of time spent delivering services using telepsychology) were included in Model 2, to determine if they added to the predictive model. As part of the procedure, tests were simultaneously conducted in SPSS to confirm if the regression assumptions were met (e.g., independence of observations, linearity, homoscedasticity, multicollinearity, significant outliers, and approximately normally distributed residuals) [72]. 

## 3. Results

The invitation to participate in the study was accepted by *N* = 310 respondents. Of these, 210 agreed to the statement of informed consent and self-identified as adults, currently working in the U.S. as an MHP. After reviewing all cases, 66 were disqualified due to pre-licensure status (i.e., trainees, registered interns), leaving a final sample of *N* = 144. The average time to take the survey was seven minutes.

### 3.1. General Characteristics of the Sample

Approximately half of participants (51%) were licensed marriage and family therapists, followed by professional counselors/clinical counselors (17%), clinical social workers (15%), and psychologists (7%). Participants were also asked to indicate their gender identity and were given the option to choose all that applied to them. Gender is a psychosocial construct distinct from sex, used to describe individual identity, expression, roles, norms, behaviors, and perceptions [73]. A majority (90%) self-identified as a woman, while approximately 8% (*n* = 11) self-identified as a man. Others were transgender, non-binary/non-conforming woman (*n* = 1), woman, non-binary/non-conforming (*n* = 2), and non-binary/non-conforming (*n* = 1). Although an option for “Prefer not to respond” was provided, it was not selected by any respondent. Most reported their highest level of education as Master of Arts/Master of Science (64.6%) or Master of Social Work (17.4%), while 17 (11.9%) had achieved a doctorate and 1 was a medical doctor practicing as a psychiatrist. The range of years of experience as a practicing MHP was from 2 to 38 (*M* = 12.03, *SD* = 7.82). The range for the average number of hours worked per week was from 2 to 50 h (*M* = 30.39, *SD* = 11.78); however, the most frequent response (*n* = 25) was 40 h per week. Most (*n* = 69) worked in private practice, with fewer (*n* = 39) practicing in an agency or community health setting or at a hospital (*n* = 7). Participants were also asked about the percentage of time they typically spend delivering mental health services via telepsychology. Relatively few (*n* = 16, 11%) reported none, while more than a third (*n* = 53, 37%) reported working 100% of the time via telepsychology.

### 3.2. Levels of Self-Compassion and Burnout

Descriptive statistics from the sample for each SCS scale and BAT scale are presented in Table 1. The highest level of self-compassion was mindfulness; the lowest was self-judgment. The highest level of burnout was psychological distress; the lowest was emotional impairment.

To provide context for the results of participants’ levels of self-compassion and burnout, frequencies were calculated according to each instrument’s cutoff values [13,65], and are presented in Table 2. Most (85%) reported moderate to high levels of self-compassion and relatively few (15%) fell into the category of likely burnout. 

### 3.3. Regression Analysis

**Figure 1:**
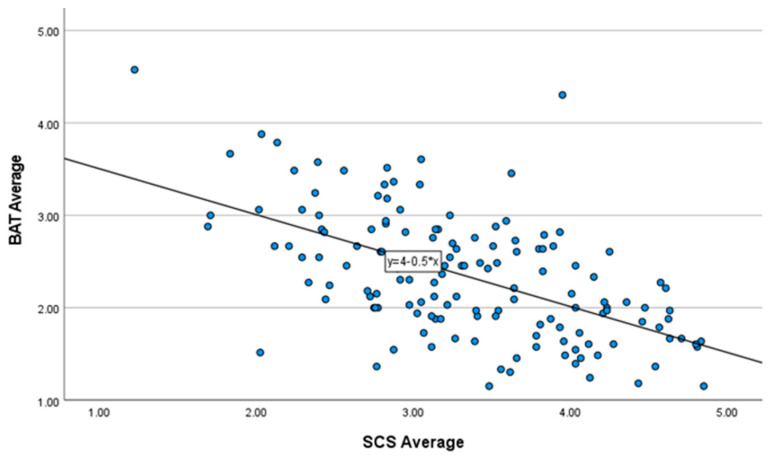
Scatterplot: Self-compassion as a predictor of burnout.

A hierarchical multiple regression was run to address the research question, and to determine if the addition of years of experience, average number of hours worked per week, and time spent in telepsychology improved the prediction of occupational burnout over self-compassion levels, alone. See Table 3 for the full details on each regression model. The linear regression for Model 1 (i.e., self-compassion as a predictor of burnout) explained approximately 31.3% of the variance in burnout; *F*(1, 135) = 61.534, *p* < 0.001, *R*^2^ = 0.313 with a large effect size (*f*^2^ = 0.456). Self-compassion level was a statistically significant negative predictor of burnout; β = −0.497, *t*(143) = -8.206, *p* < 0.001. Respondents who scored higher on the SCS reported fewer symptoms of occupational burnout. The overall SCS average was *M* = 3.33, *SD* = 0.77. The overall BAT average was *M* = 2.35, *SD* = 0.67. See Figure 1 for a scatterplot of the relationship between SCS and BAT.

The linear regression for the full model (Model 2) was statistically significant, at *R*^2^ = 0.368, *F*(4, 132) = 19.198, *p* < 0.001; adjusted *R*^2^ = 0.349 with effect size attributable to the addition of Model 2: 0.087. Adding years of experience, average number of hours worked per week, and time spent in telepsychology to the prediction of occupational burnout (Model 1) led to a modest increase in *R*^2^ of 0.055. However, only the average number of hours worked per week was a significant positive predictor; β = 0.012, *t*(136) = 2.710, *p* = 0.008. Neither years of MHP experience, β = −0.009, *t*(136) = −1.499, *p* = 0.136, nor percentage of time spent in telepsychology, β = 0.001, *t*(136) = 1.094, *p* = 0.276, were significant. The overall mean for years of experience was *M* = 12.03, *SD* = 7.82. The overall mean for hours worked per week was *M* = 30.39, *SD* = 11.78. The overall mean for the percentage of time spent in telepsychology was *M* = 66.12, *SD* = 37.64. 

Six assumptions were assessed using SPSS, to help evaluate the validity and reliability of the multiple regression analysis. Linearity was assessed using partial regression plots and a plot of studentized residuals against the predicted values [72]. No violation was detected. There was independence of the residuals, as assessed using a Durbin-Watson statistic of 1.683. Homoscedasticity was assessed with a visual inspection of a plot of studentized residuals versus unstandardized predicted values. There was no evidence of multicollinearity (i.e., no tolerance values greater than 0.1). There was only one studentized deleted residual greater than ±3 *SD* (BAT average = 4.30), and this case was retained in the analysis. Leverage points were assessed by determining if there were any problematic values (based on criterion ≥ 0.5) [72]. Fourteen cases > 5 were detected, and further analysis was conducted using ordered Cook’s Distance values to determine if any of these were highly influential (based on criterion > 1) [72]. The range of Cook’s Distance values was 0.000 to 0.142 (i.e., none > 1 were observed). For this reason, it was deemed that the assumption for the leverage values was sufficiently passed. The assumption of the normality of the residuals was also met, based on a visual inspection of a histogram with a superimposed normal curve. A Normal P-P plot of the regression standardized residuals was generated; the points were well-aligned along the diagonal line, and they appeared to be approximately normally distributed [72]. 

### 3.4. Scale Statistics

Finally, the reliability analyses were conducted in SPSS to assess the internal consistency of the 26-item SCS [53] and the 33-item BAT [7]. Both measures utilize a 5-point Likert scale. Both scales had very high levels of internal consistency [66]. Scale statistics for this study are presented in Table 4 and Table 5 alongside reliability statistics provided by the authors that resulted from psychometric testing during the scale development.

## 4. Discussion

This correlational study explored the relationship between self-compassion and burnout for currently practicing MHPs in the U.S. The results demonstrate that levels of burnout are lower for MHPs with higher levels of self-compassion (*p* < 0.001). According to the regression analysis, the predicted level of burnout decreases by approximately −0.492 (Model 1) and −0.451 (Model 2) for every one-unit increase in self-compassion. This change is not insignificant given that the full range of possible BAT scores is 1 to 5. Interestingly, the effect sizes were large, even though self-compassion levels for MHPs in this study were generally high (only 15% were in the low self-compassion category) while overall, levels of burnout were low (69% were in the no burnout category). 

This study adds to the growing body of evidence that supports the efficacy of self-compassion-based interventions for well-being [58,74,75,76], and emotional health [77]. Mindful self-compassion practices promote adaptive coping skills [16] because they involve self-awareness and treating oneself with care and understanding, rather than harsh judgment [49]. The positive correlation between well-being, self-care, mindfulness, and self-compassion is well-established [51], but not necessarily for MHPs. We propose that the results of this study indicate that individual MHPs may utilize self-compassion practices as a type of self-administered preventative self-care action against burnout [36]. MHPs can utilize mindful self-compassion practices as self-care, to direct the same level of compassion towards themselves that they show to their clients [50]. Moreover, self-compassion may be utilized at all stages of one’s career to help restore or increase well-being [16,37]. This study further supports the need for self-compassion training for MHPs, since research shows it is effective for reducing burnout symptoms [54].

We also found that, as might be expected, there was a positive correlation between the average number of hours worked per week and levels of burnout. Prior research has shown that hours of overtime are a significant predictor of burnout for psychological therapist practitioners in the U.K. [78]. MHPs in our study reported working an average of 30.39 h per week (with a range of ranging 2 to 50 h). Almost 17% reported working more than 40 h. According to the regression analysis, the predicted level of burnout increases by approximately 0.012 for each extra hour worked, a small but statistically significant difference (*p* = 0.008). Furthermore, MHPs who practice telepsychology often work from home, which leads to blurred work/life boundaries and longer work hours [79]. This provided the impetus for including the number of hours worked per week and the percentage of time spent delivering mental health services via telepsychology in the second regression model. We wanted to see if the addition of these factors improved the prediction of burnout as compared to self-compassion alone. The result for telepsychology was not significant (*p* = 0.276). However, it is worth noting that 37% of the MHPs in this study reported working 100% of the time via telepsychology, while only 11% worked exclusively in person. 

Years of experience was not a significant predictor of burnout (*p* = 0.136), which contradicts prior research that less experienced MHPs are more likely to suffer from it, possibly because they do not have the necessary training or resources to meet the challenges of the profession [6,47]. The reason for this finding is not clear; however, the distribution for the variable Years of Experience was slightly positively skewed, with about half (49%) reporting ≤9 years of experience and only 5% who reported ≥30 years of experience). 

An additional strength of this study was that the SCS reliability statistics from the sample were comparable to results obtained during the scale’s development and validation [65]. Although the BAT reliability statistics for total core burnout symptoms and the exhaustion subscale were comparable to the authors’ test samples [7], they were lower for mental distance, emotional impairment, and cognitive impairment. No scale items were modified, but possible explanations for these discrepancies are differences in sample characteristics (including cultural norms) or random variation due to the smaller sample size as compared to the Dutch and Flemish test samples, or social desirability bias, which should be considered given the low levels of burnout and high self-compassion reported by participants.

### 4.1. Study Limitations

This research methodology was nonexperimental, observational, and naturalistic. Respondents were not randomly assigned to experimental groups and no determinations of causation are feasible [80]. There may also be other explanations for the findings due to missing variables not included in this study [81]. If an alternative explanation exists, due to unaccounted for variables or confounding covariates, this could limit internal validity [82] or result in a biased estimate of the effect size [83]. The accuracy of the data is also contingent on respondents’ veracity with the self-reported data, and possibly their psychological and/or physical state while taking the survey [84]. Sizeable deviations between actual attitudes and behavior and self-reported data can limit a measures’ reliability or validity [85]. Some caution may be warranted in interpreting the results of the multiple regression analysis, since several potentially high leverage points were detected in the data during the assumption tests. These cases were examined, and none were removed based on acceptable Cook’s Distance values < 1 [72], but it is possible that there was some impact on the model parameter estimates that reduced the predictive accuracy [86]. 

Other potential limitations are the relatively small sample size (*n* = 144), and that approximately half of participants (51%) were licensed marriage and family therapists, and the majority (*n* = 129, 90%) reported their gender as “woman.” This may be attributable to the nonrandom convenience snowball sampling method, whereby participants were encouraged to forward the invitation to the study to other potentially willing respondents. This practice promotes momentum with initial contacts and may facilitate achieving the target sample size [87]; however, the seemingly overrepresentation of these groups could have resulted from sampling error (which could also be due to the relatively small sample size) or selection bias [88]. It is also possible that there was nonresponse bias, such that a significant portion of MHPs with other types of licenses elected to not respond to the survey or to not complete it, possibly due to the variables of interest, time constraints, or survey fatigue [89]. This is difficult to ascertain, but it is possible that this explains the underrepresentation of other subgroups of MHPs. Either way, this could limit the generalizability of the findings. Data collection was also intentionally restricted to licensed MHPs currently practicing in the U.S., which may limit external validity and transferability of the results to other geographic regions or populations.

### 4.2. Recommendations for Practice and Future Research

This study supports the need for more educational and occupational training opportunities to instruct MHPs on self-compassion practices, and how they are an effective self-care prevention strategy against burnout. It is imperative that clinicians recognize their personal responsibility for combating burnout; however, organizations also have a responsibility to facilitate the well-being of MHPs. Multiple factors contribute to the successful training of health care practitioners, including the need to understand the effects of burnout and how to mitigate it [43]. Teaching self-care strategies for avoiding burnout should begin as early as graduate school [38], since burnout persists in the absence of an intervention [90], and clinical training programs should prepare MHPs to approach self-care proactively [33]. This aligns with prior recommendations that there is likely a benefit to adding education on mindfulness practices to counselor training programs [91]. Afterward, education about the benefits of cultivating self-compassion should be offered through continuing education programs on psychology. 

Continuous research is also needed to assess the effects of telepsychology on MHPs since the prevalence of telehealth services continues to rise. As of 2021, 38% of Americans report that they have used technology for a visit with a medical or mental health professional, a 7% increase since the fall of 2020 [92]. This indicates that the trend toward virtual mental health services is likely to persist even after the COVID-19 pandemic. Telepsychology tends to increase the number of hours that MHPs work [79], and consequently increases the risk of burnout [78]. According to the American Psychological Association, as of October 2022, 50% of psychologists now offer in-person and virtual services, and telehealth will continue to have a significant role across the health care industry in the U.S. [93]. This will necessitate ongoing research about its effects on MHP well-being, in addition to patient well-being.

In future research, the sampling could include MHPs in the prelicensure stage or the new shorter version of the BAT [94] could be utilized. More sociodemographic variables could be added to assess group differences. The long-term effects of self-compassion on burnout could be studied using longitudinal measurement (i.e., within group research designs) to explore how MHP burnout changes over time or as the result of training. Repeated measures could further help assess differences in patient satisfaction, employee retention, or organizational functioning after self-compassion training. This would align with current developments in research in the field of organizational psychology which has seen an increase in studies on the effects of self-compassion practices in the workplace [95]. More complex research designs, such as path analysis or structural equation modeling, could help clarify the direction and magnitude of the relationships between burnout and self-compassion to allow for causal inference [96]. 

## 5. Conclusions

In summary, cultivating self-compassion appears to be a pragmatic self-care strategy for MHPs to mitigate the negative effects of burnout. We propose that self-compassion practice is a straightforward, comprehensible, economical intervention that has the added benefits of being pan-gender, pan-cultural, and appropriate for MHPs at any age or career stage. More educational and occupational training in self-compassion practices should be provided to help protect the physical and emotional well-being of MHPs. The deleterious systemic effects of burnout make MHP self-care an ethical issue, along with the need to identify protective factors, prevention, and treatment of burnout. 

## Figures and Tables

**Table 1:** SCS Scale and BAT Scale Descriptive Statistics.

Scale	*M*	*SD*	Min.	Max.
Total SCS	3.34	0.77	1.23	4.85
Self-kindness	3.36	0.85	1.20	5.00
Self-judgment	3.15	0.96	1.00	5.00
Common Humanity	3.45	0.90	1.50	5.00
Isolation	3.20	1.02	1.00	5.00
Mindfulness	3.68	0.80	1.25	5.00
Over-identification	3.21	0.93	1.00	5.00
Total BAT	2.34	0.67	1.15	4.58
Exhaustion	2.83	0.94	1.00	5.00
Mental Distance	2.00	0.81	1.00	5.00
Cognitive Impairment	2.20	0.82	1.00	4.80
Emotional Impairment	1.62	0.66	1.00	5.00
Psychological Distress	2.84	0.95	1.00	4.80
Psychosomatic Distress	2.25	0.80	1.00	4.20

**Table 2:** BAT and SCS Cutoff Score Sample Statistics.

Scale	Cutoff Values	*n* (%)
SCS		
Low self-compassion	1.0–2.5	22 (15.3)
Moderate self-compassion	2.5–3.5	61 (42.4)
High self-compassion	3.5–5.0	61 (42.4)
BAT		
Green (No burnout)	≤2.58	100 (69.4)
Orange (At risk of burnout)	2.59 to 3.01	23 (16.0)
Red (Likely burnout)	≥3.02	21 (14.6)

**Table 3:** Hierarchical Regression Results for Burnout Assessment Tool Scores.

		95% CI for *B*					
	*B*	*LL*	*UL*	*SE B*	β	*p*	*R* ^2^
Model 1						<0.001	0.313
Constant	30.990	30.566	40.414	0.214		<0.001	
Self-compassion	−0.492	−0.617	−0.368	0.063	−0.560	<0.001	
Model 2						<0.001	0.368
Constant	30.516	20.941	40.091	0.291		<0.001	
Self-compassion	−0.451	−0.576	−0.327	0.063	−0.513	<0.001	
Years of experience	−0.009	−0.022	0.003	0.006	−0.110	0.136	
Hrs. Worked/Week	0.012	0.003	0.020	0.004	0.205	0.008	
Telepsychology	0.001	−0.001	0.004	0.001	0.081	0.276	

Note. Dependent Variable: BAT average. *B* = unstandardized regression coefficient; CI = confidence interval; *LL* = lower limit; *UL* = upper limit; *SE B* = standard error of the coefficient; β = standardized coefficient. Cases were excluded listwise.

**Table 4:** SCS scale reliability statistics.

SCS Scale	Scale Items	SCS Test Sample	U.S. MHP Sample
Total	1–26	0.92	0.95
Self-kindness	5, 12, 19, 23, 26	0.78	0.86
Self-judgment	1, 8, 11, 16, 21	0.77	0.87
Common humanity	3, 7, 10, 15	0.80	0.80
Isolation	4, 13, 18, 25	0.79	0.84
Mindfulness	9, 14, 17, 22	0.75	0.82
Over-identification	2, 6, 20, 24	0.81	0.81

**Table 5:** BAT scale reliability statistics.

BAT Scale	Scale Items	Flanders Sample	Netherlands Sample	U.S. MHP Sample
Total core symptoms	1–23	0.96	0.97	0.95
Exhaustion	1–8	0.92	0.94	0.92
Mental distance	9–13	0.91	0.93	0.82
Emotional impairment	14–18	0.90	0.94	0.83
Cognitive impairment	19–23	0.92	0.94	0.90
Total secondary symptoms	24–33	0.89	0.94	0.82
Psychological complaints	24–28	*	*	0.82
Psychosomatic complaints	29–33	*	*	0.76

* Reliability statistics not available for secondary symptoms scales.

## Data Availability

The data presented in this study are available on request from the corresponding author.

## References

[1] World Health Organization Burn-Out an “Occupational Phenomenon”: International Classification of Diseases. https://www.who.int/news/item/28-05-2019-burn-out-an-occupational-phenomenon-international-classification-of-diseases.

[2] Maslach C., Schaufeli W.B., Leiter M.P. (2001). Job Burnout. Annu. Rev. Psychol..

[3] Maslach C., Jackson S.E. (1981). The Measurement of Experienced Burnout. J. Organ. Behav..

[4] Schaufeli W., De Witte H. (2017). Work Engagement in Contrast to Burnout: Real or Redundant?. Burn. Res..

[5] Jackson S.E., Maslach C. (1982). After-Effects of Job-Related Stress: Families as Victims. J. Organ. Behav..

[6] Leiter M.P., Maslach C. (1988). The Impact of Interpersonal Environment on Burnout and Organizational Commitment. J. Organ. Behav..

[7] Schaufeli W.B., Desart S., De Witte H. (2020). Burnout Assessment Tool (BAT)—Development, Validity, and Reliability. Int. J. Environ. Res. Public Health.

[8] Brenninkmeijer V. (2003). How to Conduct Research on Burnout: Advantages and Disadvantages of a Unidimensional Approach in Burnout Research. Occup. Environ. Med..

[9] McCormack H.M., MacIntyre T.E., O’Shea D., Herring M.P., Campbell M.J. (2018). The Prevalence and Cause(s) of Burnout among Applied Psychologists: A Systematic Review. Front. Psychol..

[10] Maslach C., Leiter M.P. (2016). Understanding the Burnout Experience: Recent Research and Its Implications for Psychiatry. World Psychiatry.

[11] Nadon L., De Beer L.T., Morin A.J.S. (2022). Should Burnout Be Conceptualized as a Mental Disorder?. Behav. Sci..

[12] Schaufeli W., De Witte H., Krägeloh C.U., Alyami M., Medvedev O.N. (2023). Burnout Assessment Tool (BAT): A Fresh Look at Burnout. International Handbook of Behavioral Health Assessment.

[13] Schaufeli W.B., De Witte H., Hakanen J.J., Kaltiainen J., Kok R. (2023). How to Assess Severe Burnout? Cutoff Points for the Burnout Assessment Tool (BAT) Based on Three European Samples. Scand. J. Work Environ. Health.

[14] Mental Health Practitioner Definition. https://www.lawinsider.com/dictionary/mental-health-practitioner.

[15] Yang Y., Hayes J.A. (2020). Causes and Consequences of Burnout among Mental Health Professionals: A Practice-Oriented Review of Recent Empirical Literature. Psychotherapy.

[16] O’Connor K., Muller Neff D., Pitman S. (2018). Burnout in Mental Health Professionals: A Systematic Review and Meta-Analysis of Prevalence and Determinants. Eur. Psychiatr..

[17] Springer Publishing Company (2023). Managing Stress during Your Counseling Practicum/Internship.

[18] Reed K., Cochran K.L., Edelblute A., Manzanares D., Sinn H., Henry M., Moss M. (2020). Creative Arts Therapy as a Potential Intervention to Prevent Burnout and Build Resilience in Health Care Professionals. AACN Adv. Crit. Care.

[19] Simionato G.K., Simpson S. (2018). Personal Risk Factors Associated with Burnout among Psychotherapists: A Systematic Review of the Literature. J. Clin. Psychol..

[20] West C.P., Dyrbye L.N., Sloan J.A., Shanafelt T.D. (2009). Single Item Measures of Emotional Exhaustion and Depersonalization Are Useful for Assessing Burnout in Medical Professionals. J. Gen. Intern. Med..

[21] Byrne Z.S., Peters J.M., Weston J.W. (2016). The Struggle with Employee Engagement: Measures and Construct Clarification Using Five Samples. J. Appl. Psychol..

[22] Taris T.W., Ybema J.F., Beek I.V. (2017). Burnout and Engagement: Identical Twins or Just Close Relatives?. Burn. Res..

[23] Edú-Valsania S., Laguía A., Moriano J.A. (2022). Burnout: A Review of Theory and Measurement. Int. J. Environ. Res. Public Health.

[24] Shoji K., Cieslak R., Smoktunowicz E., Rogala A., Benight C.C., Luszczynska A. (2016). Associations between Job Burnout and Self-Efficacy: A Meta-Analysis. Anxiety Stress Coping.

[25] Ekman E., Halpern J. (2015). Professional Distress and Meaning in Health Care: Why Professional Empathy Can Help. Soc. Work. Health Care.

[26] Scanlan J.N., Still M. (2019). Relationships between Burnout, Turnover Intention, Job Satisfaction, Job Demands and Job Resources for Mental Health Personnel in an Australian Mental Health Service. BMC Health Serv. Res..

[27] Wood A.E., Prins A., Bush N.E., Hsia J.F., Bourn L.E., Earley M.D., Walser R.D., Ruzek J. (2017). Reduction of Burnout in Mental Health Care Providers Using the Provider Resilience Mobile Application. Community Ment. Health J..

[28] Auerbach J., Miller B.F. (2020). COVID-19 Exposes the Cracks in Our Already Fragile Mental Health System. Am. J. Public Health.

[29] Bressi S.K., Vaden E.R. (2017). Reconsidering Self Care. Clin. Soc. Work J..

[30] Crowder R., Sears A. (2017). Building Resilience in Social Workers: An Exploratory Study on the Impacts of a Mindfulness-Based Intervention. Aust. Soc. Work.

[31] Narasimhan M., Kapila M. (2019). Implications of Self-Care for Health Service Provision. Bull. World Health Organ..

[32] Norcross J.C., Phillips C.M. (2020). Psychologist Self-Care during the Pandemic: Now More than Ever. J. Health Serv. Psychol..

[33] Posluns K., Gall T.L. (2020). Dear Mental Health Practitioners, Take Care of Yourselves: A Literature Review on Self-Care. Int. J. Adv. Couns..

[34] Wei H., Kifner H., Dawes M.E., Wei T.L., Boyd J.M. (2020). Self-Care Strategies to Combat Burnout among Pediatric Critical Care Nurses and Physicians. Crit. Care Nurse.

[35] Dattilio F.M. (2015). The Self-Care of Psychologists and Mental Health Professionals: A Review and Practitioner Guide. Aust. Psychol..

[36] Pakenham K.I. (2015). Comment on “The Self-Care of Psychologists and Mental Health Professionals” (Dattilio, 2015). Aust. Psychol..

[37] Butts C.M., Gutierrez D. (2018). Dispositional Mindfulness and Personal Distress as Predictors of Counseling Self-Efficacy. Couns. Educ. Superv..

[38] Rudaz M., Twohig M.P., Ong C.W., Levin M.E. (2017). Mindfulness and Acceptance-Based Trainings for Fostering Self-Care and Reducing Stress in Mental Health Professionals: A Systematic Review. J. Context. Behav. Sci..

[39] Butler L.D., Mercer K.A., McClain-Meeder K., Horne D.M., Dudley M. (2019). Six Domains of Self-Care: Attending to the Whole Person. J. Hum. Behav. Soc. Environ..

[40] Edwards J.L., Crisp D.A. (2017). Seeking Help for Psychological Distress: Barriers for Mental Health Professionals. Aust. J. Psychol..

[41] Kissil K., Niño A. (2017). Does the Person-of-the-Therapist Training (POTT) Promote Self-Care? Personal Gains of MFT Trainees Following POTT: A Retrospective Thematic Analysis. J. Marital. Fam. Ther..

[42] Prasada K.D.V., Vaidyab R.W., Mangipudic M.R. (2020). Effect of Occupational Stress and Remote Working on Psychological Well-Being of Employees: An Empirical Analysis during COVID-19 Pandemic Concerning Information Technology Industry in Hyderabad. Indian J. Commer. Manag. Stud..

[43] Rokach A., Boulazreg S. (2022). The COVID-19 Era: How Therapists Can Diminish Burnout Symptoms through Self-Care. Curr. Psychol..

[44] Creswell J.D. (2017). Mindfulness Interventions. Annu. Rev. Psychol..

[45] De Vibe M., Solhaug I., Rosenvinge J.H., Tyssen R., Hanley A., Garland E. (2018). Six-Year Positive Effects of a Mindfulness-Based Intervention on Mindfulness, Coping and Well-Being in Medical and Psychology Students; Results from a Randomized Controlled Trial. PLoS ONE.

[46] Nelson J.R., Hall B.S., Anderson J.L., Birtles C., Hemming L. (2018). Self–Compassion as Self-Care: A Simple and Effective Tool for Counselor Educators and Counseling Students. J. Creat. Ment. Health.

[47] Sonnentag S. (2005). Burnout Research: Adding an off-Work and Day-Level perspective. Work Stress.

[48] Lomas T., Medina J.C., Ivtzan I., Rupprecht S., Eiroa-Orosa F.J. (2018). A Systematic Review of the Impact of Mindfulness on the Well-Being of Healthcare Professionals. J. Clin. Psychol..

[49] Beer O.W., Phillips R., Stepney L., Quinn C.R. (2020). OUP Accepted Manuscript. Br. J. Soc. Work.

[50] Coaston S.C. (2017). Self-Care Through Self-Compassion: A Balm for Burnout. Prof. Couns..

[51] Germer C.K., Neff K.D. (2013). Self-Compassion in Clinical Practice: Self-Compassion. J. Clin. Psychol..

[52] Lakioti A., Stalikas A., Pezirkianidis C. (2020). The Role of Personal, Professional, and Psychological Factors in Therapists’ Resilience. Prof. Psychol. Res. Pract..

[53] Neff K. (2003). Self-Compassion: An Alternative Conceptualization of a Healthy Attitude toward Oneself. Self Identity.

[54] Eriksson T., Germundsjö L., Åström E., Rönnlund M. (2018). Mindful Self-Compassion Training Reduces Stress and Burnout Symptoms among Practicing Psychologists: A Randomized Controlled Trial of a Brief Web-Based Intervention. Front. Psychol..

[55] Neff K.D., Tóth-Király I., Knox M.C., Kuchar A., Davidson O. (2021). The Development and Validation of the State Self-Compassion Scale (Long- and Short Form). Mindfulness.

[56] Jaarsma T., Strömberg A., Dunbar S.B., Fitzsimons D., Lee C., Middleton S., Vellone E., Freedland K.E., Riegel B. (2020). Self-Care Research: How to Grow the Evidence Base?. Int. J. Nurs. Stud..

[57] Eller L.S., Lev E.L., Yuan C., Watkins A.V. (2018). Describing Self-Care Self-Efficacy: Definition, Measurement, Outcomes, and Implications: Describing Self-Care Self-Efficacy. Int. J. Nurs. Termin. Knowl..

[58] Neff K.D., Costigan A. (2014). Self-Compassion, Wellbeing, and Happiness. Psychol. Osterr..

[59] Bluth K., Eisenlohr-Moul T.A. (2017). Response to a Mindful Self-compassion Intervention in Teens: A within-Person Association of Mindfulness, Self-Compassion, and Emotional Well-Being Outcomes. J. Adolesc..

[60] Olson K., Kemper K.J. (2014). Factors Associated with Well-Being and Confidence in Providing Compassionate Care. J. Evid. Based Complement. Altern. Med..

[61] Sharifi M., Asadi-Pooya A.A., Mousavi-Roknabadi R.S. (2020). Burnout among Healthcare Providers of COVID-19; a Systematic Review of Epidemiology and Recommendations: Burnout in Healthcare Providers. Arch. Acad. Emerg. Med..

[62] Sinclair S., Kondejewski J., Raffin-Bouchal S., King-Shier K.M., Singh P. (2017). Can Self-Compassion Promote Healthcare Provider Well-Being and Compassionate Care to Others? Results of a Systematic Review. Appl. Psychol. Health Well-Being.

[63] Faul F., Erdfelder E., Buchner A., Lang A.-G. (2009). Statistical Power Analyses Using G*Power 3.1: Tests for Correlation and Regression Analyses. Behav. Res. Methods.

[64] Faul F., Erdfelder E., Lang A.-G., Buchner A. (2007). G*Power 3: A Flexible Statistical Power Analysis Program for the Social, Behavioral, and Biomedical Sciences. Behav. Res. Methods.

[65] Neff K.D. (2003). The Development and Validation of a Scale to Measure Self-Compassion. Self Identity.

[66] Nunnally J.C., Bernstein I.H. (2010). Psychometric Theory.

[67] Azizi A., Mohammadkhani P., Foroughi A.A., Lotfi S., Bahramkhani M. (2013). The Validity and Reliability of the Iranian Version of the Self-Compassion Scale. Pract. Clin. Psychol..

[68] Chen J., Yan L., Zhou L. (2011). Reliability and Validity of Chinese Version of Self-compassion Scale. Chin. J. Clin. Psychol..

[69] American Psychological Association Ethical Principles of Psychologists and Code of Conduct. https://www.apa.org/ethics/code.

[70] IBM SPSS Statistics. https://www.ibm.com/products/spss-statistics.

[71] Corrales D., Corrales J., Ledezma A. (2018). How to Address the Data Quality Issues in Regression Models: A Guided Process for Data Cleaning. Symmetry.

[72] Laerd Statistics Multiple Regression Using SPSS Statistics. Statistical Tutorials and Software Guides.

[73] Tadiri C.P., Raparelli V., Abrahamowicz M., Kautzy-Willer A., Kublickiene K., Herrero M.-T., Norris C.M., Pilote L. (2021). Methods for Prospectively Incorporating Gender into Health Sciences Research. J. Clin. Epidemiol..

[74] Ferrari M., Hunt C., Harrysunker A., Abbott M.J., Beath A.P., Einstein D.A. (2019). Self-Compassion Interventions and Psychosocial Outcomes: A Meta-Analysis of RCTs. Mindfulness.

[75] Beaumont E., Durkin M., Hollins Martin C.J., Carson J. (2016). Measuring Relationships between Self-Compassion, Compassion Fatigue, Burnout and Well-Being in Student Counsellors and Student Cognitive Behavioural Psychotherapists: A Quantitative Survey. Couns. Psychother. Res..

[76] George L., Wallace J.C., Snider J.B., Suh H. (2023). Self-Compassion, Performance, and Burnout: Surfacing an Unknown Work Construct. Group Organ. Manag..

[77] Luo X., Qiao L., Che X. (2018). Self-Compassion Modulates Heart Rate Variability and Negative Affect to Experimentally Induced Stress. Mindfulness.

[78] Westwood S., Morison L., Allt J., Holmes N. (2017). Predictors of Emotional Exhaustion, Disengagement and Burnout among Improving Access to Psychological Therapies (IAPT) Practitioners. J. Ment. Health.

[79] MacDonald L.M.-H. (2022). Impact of Working from Home on Addressing Practitioner Burnout and Work–Life Balance in Mental Health. Perm. J..

[80] Creswell J.W., Creswell J.D. (2018). Research Design: Qualitative, Quantitative, and Mixed Methods Approaches.

[81] Trochim W.M.K., Donnelly J.P., Arora K. (2016). Research Methods: The Essential Knowledge Base.

[82] Rohrer J.M. (2018). Thinking Clearly About Correlations and Causation: Graphical Causal Models for Observational Data. Adv. Methods Pract. Psychol. Sci..

[83] Kashner T.M., Henley S.S., Golden R.M., Zhou X.-H. (2020). Making Causal Inferences about Treatment Effect Sizes from Observational Datasets. Biostat. Epidemiol..

[84] Jackson S.L. (2015). Research Methods and Statistics: A Critical Thinking Approach.

[85] Prior M. (2009). The Immensely Inflated News Audience: Assessing Bias in Self-Reported News Exposure. Public Opin. Q..

[86] Field A., Seaman J. (2018). Discovering Statistics Using IBM® SPSS® Statistics.

[87] Parker C., Scott S., Geddes A. (2020). Snowball Sampling. SAGE Research Methods Foundations.

[88] Greenacre Z.A. (2016). The Importance of Selection Bias in Internet Surveys. Open J. Stat..

[89] Wright G. (2015). An Empirical Examination of the Relationship between Nonresponse Rate and Nonresponse Bias. Stat. J. IAOS.

[90] Schabram K., Heng Y.T. (2021). How Other- and Self-Compassion Reduce Burnout through Resource Replenishment. Acad. Manag. J..

[91] Zhang L., Ren Z., Jiang G., Hazer-Rau D., Zhao C., Shi C., Lai L., Yan Y. (2021). Self-Oriented Empathy and Compassion Fatigue: The Serial Mediation of Dispositional Mindfulness and Counselor’s Self-Efficacy. Front. Psychol..

[92] New Nationwide Poll Shows an Increased Popularity for Telehealth Services. https://www.psychiatry.org:443/news-room/news-releases/new-nationwide-poll-shows-an-increased-popularity.

[93] Telehealth Is Here to Stay. Psychologists Should Equip Themselves to Offer It. https://www.apa.org/monitor/2022/10/future-of-telehealth.

[94] Hadžibajramović E., Schaufeli W., De Witte H. (2020). A Rasch Analysis of the Burnout Assessment Tool (BAT). PLoS ONE.

[95] Dodson S.J., Heng Y.T. (2022). Self-compassion in Organizations: A Review and Future Research Agenda. J. Organ. Behav..

[96] Cunningham S. (2021). Causal Inference: The Mixtape.

